# Supplementary material for: Genomics of experimental adaptation of Staphylococcus aureus to a natural combination of insect antimicrobial peptides
Source: Sci Rep. 2018 Oct 18;8:15359. doi: 10.1038/s41598-018-33593-7 (PMC6193990; doi:10.1038/s41598-018-33593-7)
Supplement: Supplementary file 2 — Supplementary Table 2 [file 41598_2018_33593_MOESM2_ESM.pdf]

Table S2. Cross resistance against antibiotics

type	line	Chloramphenicol	Kanamycin	Ampicillin	Ciprofloxacin	Tetracycline	Gentamicin	Erythromycin	Rifampicin
colony	T1-1-L-C	4	8	0.25	0.25	2	0.25	0.25	MIC<0,0625
colony	T1-1-S-C	8	2	0.125	0.25	0.5	0.125	0.25	MIC<0,0625
colony	T1-2-L-C	4	4	0.125	0.25	0.5	0.125	0.125	MIC<0,0625
colony	T1-2-S-C	4	8	0.25	0.5	1	0.125	0.25	MIC<0,0625
colony	T1-3-L-C	4	2	0.25	0.125	0.5	0.125	0.25	MIC<0,0625
colony	T1-3-S-C	8	4	0.125	0.25	0.5	0.125	0.25	MIC<0,0625
colony	T1-4-C	4	4	0.25	0.0625	0.5	0.125	0.125	MIC<0,0625
colony	T1-5-C	8	4	0.125	0.5	1	MIC<0,0625	0.25	MIC<0,0625
colony	T1T2-1-C	8	16	0.25	0.25	0.5	0.5	0.5	MIC<0,0625
colony	T1T2-2-C	8	8	0.125	0.5	MIC<2	0.5	0.125	MIC<0,0625
colony	T1T2-3-C	4	4	0.25	0.25	1	0.125	0.25	MIC<0,0625
colony	T1T2-4-L-C	8	8	0.25	0.5	0.5	0.5	0.5	MIC<0,0625
colony	T1T2-4-S-C	4	2	0.25	0.125	0.5	0.25	0.25	MIC<0,0625
colony	T1T2-5-L-C	8	4	0.25	1	1	0.5	0.25	MIC<0,0625
colony	T1T2-5-S-C	8	4	0.25	0.5	0.5	0.25	0.25	MIC<0,0625
colony	con-1-C	8	4	0.125	0.25	0.5	0.25	0.125	MIC<0,0625
colony	con-2-C	8	4	0.125	0.125	0.5	0.125	0.125	MIC<0,0625
colony	con-3-C	8	8	0.125	0.25	0.5	0.25	0.125	MIC<0,0625
colony	con-4-C	4	8	0.125	0.5	0.5	0.25	0.125	MIC<0,0625
colony	con-5-C	8	4	0.125	0.5	1	MIC<0,0625	0.125	MIC<0,0625
colony	ANCESTOR-[illegible]	8	4	0.25	0.5	1	0.125	0.25	MIC<0,0625
